# O.1.1-2 How can the sport sector help tackle mental health problems?

**DOI:** 10.1093/eurpub/ckad133.078

**Published:** 2023-09-11

**Authors:** Wikke van Stam

**Affiliations:** Mulier Instituut, The Netherlands; w.vanstam@mulierinstituut.nl

## Abstract

Dutch citizens' mental health is under pressure, and seems to have worsened during Covid-19. Research shows that sport and exercise can help to become and stay mentally healthy (e.g. Singh et al., 2023; Eime et al., 2013), but mentally unhealthy people currently exercise less (Ooms et al., 2019). We aimed to answer the questions: to what extent is the sport sector ready to help reduce and prevent mental health issues? And how can it be equipped for this task?

Our research focussed on sport clubs and community sport coaches (CSC; professionals who motivate inhabitants to become physically active by connecting the sport sector with other sectors). In 2022, we conducted an online questionnaire amongst a panel of Dutch sport clubs (n = 313), and one amongst a panel of CSCs (n = 130). On the obtained data, we conducted descriptive analysis. We also held focus group sessions with sport clubs (n = 3) and CSCs (n = 5).

Findings show that 5% of sport clubs pay specific attention to improving their members' mental health, and 72% of CSCs focus their work on this. All sport clubs and the vast majority of CSCs who focus on mental health, try to prevent people becoming mentally unhealthy. Only 5% of the CSCs (and no sport clubs in our research) focus on people with serious psychological complaints. Whether sport clubs and CSCs are involved with mental health often depends on the ‘feeling’ a CSC or trainer has for this topic. To equip sport clubs and CSCs to become more active on mental health, awareness (knowing they can make a difference) and a local network (to feel more competent dealing with mental health together with partners) seem to be necessary.

There is great potential for the sport sector to tackle mental health problems, both preventive and curative. However, the sector must be more equipped. Policy makers and local actors can use sport more often to tackle mental health issues and create local networks. For a broader view of the sport sector, further research on e.g. commercial and differently organized sport providers is needed.

This research is funded by the Dutch Ministry of Health, Welfare and Sport.

